# Physiological Effect of XoxG(4) on Lanthanide-Dependent Methanotrophy

**DOI:** 10.1128/mBio.02430-17

**Published:** 2018-03-27

**Authors:** Yue Zheng, Jing Huang, Feng Zhao, Ludmila Chistoserdova

**Affiliations:** a Department of Chemical Engineering, University of Washington, Seattle, Washington, USA; b CAS Key Laboratory of Urban Pollutant Conversion, Institute of Urban Environment, Chinese Academy of Sciences, Xiamen, China; c University of Chinese Academy of Sciences, Beijing, China; Oregon State University

**Keywords:** XoxG, XoxF, MxaFI, MxaG, *Methylomonas*, methanol dehydrogenase

## Abstract

A recent surprising discovery of the activity of rare earth metals (lanthanides) as enzyme cofactors as well as transcriptional regulators has overturned the traditional assumption of biological inertia of these metals. However, so far, examples of such activities have been limited to alcohol dehydrogenases. Here we describe the physiological effects of a mutation in *xoxG*, a gene encoding a novel cytochrome, XoxG(4), and compare these to the effects of mutation in XoxF, a lanthanide-dependent methanol dehydrogenase, at the enzyme activity level and also at the community function level, using *Methylomonas* sp. strain LW13 as a model organism. Through comparative phenotypic characterization, we establish XoxG as the second protein directly involved in lanthanide-dependent metabolism, likely as a dedicated electron acceptor from XoxF. However, mutation in XoxG caused a phenotype that was dramatically different from the phenotype of the mutant in XoxF, suggesting a secondary function for this cytochrome, in metabolism of methane. We also purify XoxG(4) and demonstrate that this protein is a true cytochrome *c*, based on the typical absorption spectra, and we demonstrate that XoxG can be directly reduced by a purified XoxF, supporting one of its proposed physiological functions. Overall, our data continue to suggest the complex nature of the interplay between the calcium-dependent and lanthanide-dependent alcohol oxidation systems, while they also suggest that addressing the roles of these alternative systems is essential at the enzyme and community function level, in addition to the gene transcription level.

## IMPORTANCE

The lanthanide-dependent biochemistry of living organisms remains a barely tapped area of knowledge. So far, only a handful of lanthanide-dependent alcohol dehydrogenases have been described, and their regulation by lanthanides has been demonstrated at the transcription level. Little information is available regarding the concentrations of lanthanides that could support sufficient enzymatic activities to support specific metabolisms, and so far, no other redox proteins involved in lanthanide-dependent methanotrophy have been demonstrated. The research presented here provides enzyme activity-level data on lanthanide-dependent methanotrophy in model a methanotroph. Additionally, we identify a second protein important for lanthanide-dependent a metabolism in this organism, XoxG(4), a novel cytochrome. XoxG(4) appears to have multiple functions in methanotrophy, one function as an electron acceptor from XoxF and another function remaining unknown. On the basis of the dramatic phenotype of the XoxG(4) mutant, this function must be crucial for methanotrophy.

## INTRODUCTION

Aerobic methanotrophs are major players in methane consumption in environments in which molecular oxygen is available, such as soils, lake sediments, permafrosts, and water columns (1, 2). Metabolism of methane involves its conversion to methanol, which is further oxidized to formaldehyde by methanol dehydrogenase (MDH), followed by further oxidation to CO_2_ or assimilation into biomass, via traditional methylotrophy pathways that have been established and characterized over decades (1, 3). Recently, the methanotrophy field has experienced a revolution in terms of the new outlook at the methanol oxidation step. It appears that, in addition to the well-characterized, calcium-dependent methanol dehydrogenase (the MxaFI type) (4), an alternative enzyme is encoded in methanotroph genomes, XoxF, which requires lanthanides (Ln). While Ln are typically present in a redox state of 3+, the redox state can vary for at least some of the metals (5); thus, we will not be indicating a specific redox state for Ln throughout this article. *xoxF* and *mxaF* appear to be inversely regulated, at the level of transcription, by the presence of Ln, the mechanism known as the “Ln switch” (6–10). Moreover, it appears that XoxF might be a more important MDH in nature, as in some methanotrophs, only Xox-type MDH is encoded (11, 12).

In recent years, the communal function of methanotrophs has been increasingly recognized (13). Remarkably, the Ln switch appears to also be affected dependent on whether methanotrophs are cultivated as pure cultures or as communities (14). Differential transcriptional regulation of genes for alternative MDH enzymes has also been demonstrated in the absence of added Ln (15).

Like MxaFI-type MDH, XoxF-type MDH must transfer electrons onto a cytochrome. Indeed, genes predicted to encode cytochromes *c* have been identified in the vicinity of *xoxF* genes in the genomes of alpha- and betaproteobacterial methylotrophs, but not in gammaproteobacterial methanotrophs (15). However, a highly expressed gene for a putative cytochrome has been identified in the latter, and it has been proposed to encode a dedicated cytochrome for XoxF, XoxG (15). Remarkably, XoxG cytochromes encoded by alpha-, beta-, and gammaproteobacteria are very divergent, which is in contrast with the MxaG cytochromes that are electron acceptors from MxaFI-type MDH enzymes (16, 17). The phylogeny of XoxG and MxaG proteins has been recently presented by Yu et al. (15), separating them into four unrelated groups: XoxG1/MxaG, XoxG2/MauO, XoxG3, and XoxG4, the latter being specific to gammaproteobacterial methanotrophs. This recently uncovered complexity among the cytochrome components further highlights the complexity of the Xox system, as XoxF enzymes are also very divergent and have been separated into multiple phylogenetic clades, such as gammaproteobacteria encoding the XoxF5 type (3, 15, 18, 19).

While the recent developments in understanding the differential functions of MxaFI-type versus XoxF-type MDH enzymes have been very exciting, the data on the physiological effects of the alternative enzymes remain scarce. So far, the inverse regulation by Ln has been tested mostly at the transcriptional level but not at the enzyme activity level (6–10), and thus, this important information is still missing. While the electron acceptors from XoxF have been proposed based on genome location or on expression patterns, they also remained uncharacterized. Additional unknowns include the potentially differential roles of the alternative MDH enzymes in the communal function in methane oxidation (2, 14, 15).

In this study, we addressed some of these outstanding questions by assessing the functional roles of alternative MDH enzymes, at the enzyme level, through analysis of respective knockout mutants, in a model methanotroph *Methylomonas* sp. strain LW13, demonstrating that the two alternative enzymes must both be functional under certain conditions. We also identify a cytochrome, XoxG(4), that is functionally linked to XoxF in *Methylomonas* sp. LW13 and demonstrate its complex function, with the XoxG mutant phenotype differing from the XoxF mutant phenotype. We further characterize suppressor mutants in both XoxF and XoxG backgrounds, suggestive of a strong selective pressure in the presence of added Ln. We finally evaluate the performance of the mutants in alternative MDH enzymes as parts of communities utilizing methane.

## RESULTS

### Enzyme-level demonstration of a lanthanide-mediated switch between alternative methanol dehydrogenases.

**FIG 1:**
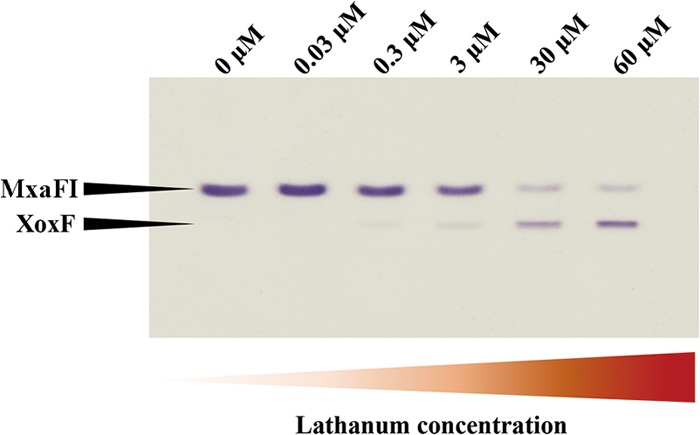
MDH activity as a function of La concentration. Alternative MDH enzymes were visualized through activity staining of the gel, after separation by electrophoresis in a gradient (4 to 25%) polyacrylamide gel. Wild-type *Methylomonas* sp. strain LW13 was grown in the presence of variable concentrations of La (0, 0.03, 0.3, 3, 30 and 60 μM), cell extracts were adjusted to approximately 3 mg/ml protein, and 10 μl of extract was applied in each lane of the gel.

A mechanism effecting inverse regulation of transcription from genes encoding alcohol dehydrogenases with alternative metal dependencies, known as the “Ln switch,” has been well documented, and low, nanomolar concentrations of Ln have been shown to trigger the switch (6, 7, 9, 10, 20, 21). However, increase in the growth rate as a function of Ln concentration has also been demonstrated, suggesting that higher concentrations of Ln must be required for optimal MDH activity (11). Ln concentration response has not yet been demonstrated at the enzyme level. Here we developed a protocol for visualizing the two alternative enzymes in *Methylomonas* sp. strain LW13. Like other gammaproteobacterial methanotrophs (7), the *Methylomonas* sp. LW13 genome encodes a single XoxF MDH (22), presenting a simple experimental model. Cultures were grown in media lacking any added Ln and in media supplemented with increasing concentrations of lanthanum (La) (0.03 to 60 μM), and activities of alternative MDH enzymes were assessed through staining of the gel (Fig. 1 and see Fig. S2A in the supplemental material). In accordance with this assay, the MxaFI enzyme retained high activity until the concentration of La reached 3 μM, and it retained significant activity at La concentrations as high as 30 to 60 μM (Fig. 1). Low activity of XoxF was detected at 0.3 μM, gradually increasing with the concentration of La. These data need to be interpreted with caution though, as the activities of MxaFI and XoxF cannot be compared directly using the artificial dye assay, as the two enzymes may have and likely do have different efficiencies in reducing these dyes. Thus, only relative activities should be discussed. Accordingly, we observed a decrease in the measured specific MDH activity with the increase in the concentration of La, reflective of the switch from MxaFI to XoxF activity (Fig. S2A).

10.1128/mBio.02430-17.1 FIG S1  Cells of *Methylomonas* sp. LW13 and M. methylotrophus Q8 can be easily distinguished via flow cytometry based on difference in size. Download FIG S1, TIF file, 2.5 MB. Copyright © 2018 Zheng et al. 2018 Zheng et al. This content is distributed under the terms of the Creative Commons Attribution 4.0 International license.

10.1128/mBio.02430-17.2 FIG S2  Lanthanide-dependent MDH activity and gene expression. (A) Specific activities of MDH measured in extracts of cells grown in the presence of different concentrations of La using the artificial dye assay. La concentrations are indicated next to each data set. (B) Relative expression of *xoxF*, *mxaF*, *mxaG*, and *xoxG4*. Expression of *xoxG4* appears not to be significantly affected. Real-time quantitative RT-PCR (qRT-PCR) assay was performed on RNA isolated from *Methylomonas* sp. LW13 cells grown in the presence or absence of 30 μM La. Values represent the means for three replicates with standard deviations. Download FIG S2, TIF file, 2.7 MB. Copyright © 2018 Zheng et al. 2018 Zheng et al. This content is distributed under the terms of the Creative Commons Attribution 4.0 International license.

The levels of *xoxF* and *mxaF* transcripts were compared, using real-time reverse transcription-PCR (RT-PCR) technology, under no La versus 30 μM La conditions, and these transcripts demonstrated inverse response to La (Fig. S2B), as previously reported for other organisms (8, 10, 20, 21). The regulation of the gene encoding the electron acceptor from MxaFI, MxaG (16, 17), followed the expression pattern of *mxaF*, while regulation of the recently identified gene, *xoxG4*, proposed to encode an electron acceptor from XoxF in gammaproteobacteria (15) did not show a strong response to La (Fig. S2B), in agreement with prior observations (15).

### Physiological effects of mutations in XoxG, XoxF, MxaG, and MxaF.

**FIG 2:**
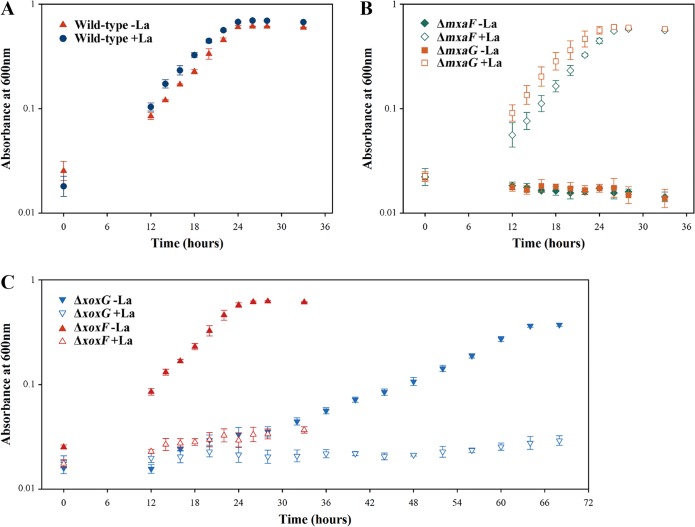
Growth curves for the wild-type strain (A), Δ*mxaF* and Δ*mxaG* mutants (B), and Δ*xoxF* and Δ*xoxG* mutants (C) grown in the presence of 30 μM La (+La) or absence of La (-La).

**FIG 3:**
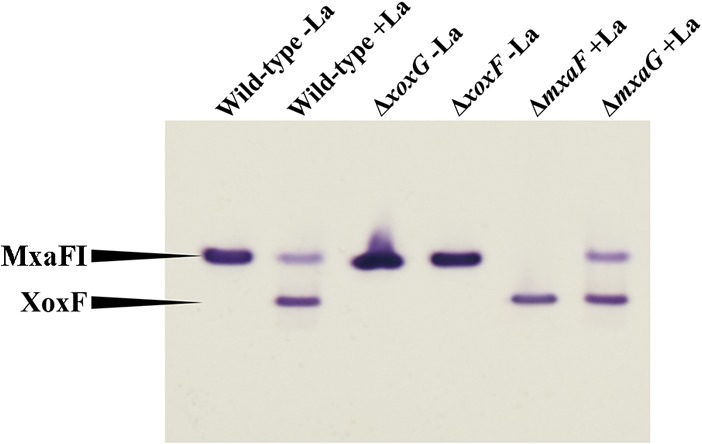
MDH activities in mutants generated in this study compared to wild-type strain activities. MDH enzymes were visualized through activity staining of the gel, after separation by electrophoresis in a gradient (4 to 25%) polyacrylamide gel. La was supplied at 30 μM where indicated. Cell extracts were adjusted to approximately 3 mg/ml protein, and 10 μl of extract was applied in each lane of the gel.

We constructed knockout mutations in *xoxG*, *xoxF*, *mxaG*, and *mxaF* and investigated growth phenotypes of the mutants. The main goals of this experiment were to establish whether XoxG is involved in Ln-dependent methanol oxidation and whether the phenotype of this mutant would be Ln responsive. Knockout *xoxG* and *xoxF* mutants were selected on La-free nitrate mineral salts (NMS) medium (23). Conversely, *mxaG* and *mxaF* mutants were selected in the presence of La, as previously reported (30 μM) (6, 7). While colonies of recombinants with insertions into *xoxF*, *mxaG*, and *mxaF* appeared on the respective selective plates after approximately 3 days, the colonies of the recombinants with insertions into *xoxG* took up to 3 weeks to appear. When tested on La-supplemented versus La-free media on plates, *xoxF*, *mxaG*, and *mxaF* mutants yielded expected phenotypes, the former being inhibited by the addition of La, and the latter two requiring Ln for growth, as previously reported for other organisms (6, 7). *xoxG* mutants displayed an Ln-responsive phenotype: while already showing impaired growth on Ln-free plates, they showed no appreciable growth on plates supplemented with La (however, see below). Growth in liquid media was also measured for all the mutants and compared to wild-type strain performance in the absence or presence of La. Δ*mxaF* and Δ*mxaG* mutants grew as well as the wild-type strain in the presence of La, but they could not grow in the absence of La, as expected (Fig. 2A and B; Table 1). The Δ*xoxF* mutant showed wild-type growth rate in the absence of La but could not grow in the presence of La (Fig. 2C). The Δ*xoxG* mutant showed significantly reduced growth in the absence of La but could not grow in the presence of La (Fig. 2C; Table 1). This identifies XoxG as the second protein/function that is subject to Ln regulation, in addition to XoxF, further suggesting its role as an electron acceptor from XoxF. However, the dramatic difference in the phenotypes of *ΔxoxF* and *ΔxoxG* mutants suggests that XoxG likely has a second function in the metabolism of *Methylomonas* sp. LW13, and this function appears to be important for both La-dependent and Ca-dependent methylotrophy. The MDH activity, as measured using the artificial dye assay, appeared somewhat elevated in the *ΔxoxG* mutant compared to other strains (Fig. 3 and S3).

**TABLE 1:** Doubling times of mutants compared to the wild type

Strain	Doubling time (h) of strain grown^a^	
	Without La	With La
Wild-type	4.75 ± 0.35	4.25 ± 0.30
Δ*xoxF* mutant	4.81 ± 0.36	
Δ*xoxG* mutant	11.86 ± 1.01*	
Δ*mxaF* mutant		3.58 ± 0.21
Δ*mxaG* mutant		3.91 ± 0.25

a Doubling time values represent the means ± standard deviations for three replicates, and they were calculated from four time points during the exponential phase of growth. La was supplied at 30 μM. The asterisk indicates a statistically highly significant change in growth rate compared to other strains (*P* < 0.001).

10.1128/mBio.02430-17.3 FIG S3  Specific activity of MDH in mutants generated in this study. Strains and conditions are shown next to each data set. La was supplied at 30 μM. Values represent the means for three replicates with standard deviations. Download FIG S3, TIF file, 2.7 MB. Copyright © 2018 Zheng et al. 2018 Zheng et al. This content is distributed under the terms of the Creative Commons Attribution 4.0 International license.

We performed transcriptome sequencing (RNA-seq) analysis comparing whole-genome gene expression between the wild type and the *ΔxoxG* mutant, and we identified a set of genes that showed statistically significant differential expression (Table S1). However, no clear clues as to the second function of XoxG4 emerged from this analysis, and thus, gene-by-gene knockout style interrogation is necessary to obtain any further leads, which will be pursued in the future.

10.1128/mBio.02430-17.8 TABLE S1  Differential gene expression between wild-type and Δ*xoxG* strains. Download TABLE S1, DOCX file, 0.1 MB. Copyright © 2018 Zheng et al. 2018 Zheng et al. This content is distributed under the terms of the Creative Commons Attribution 4.0 International license.

### Effects of mutations in XoxG, XoxF, MxaG, and MxaF on communal behavior.

**FIG 4:**
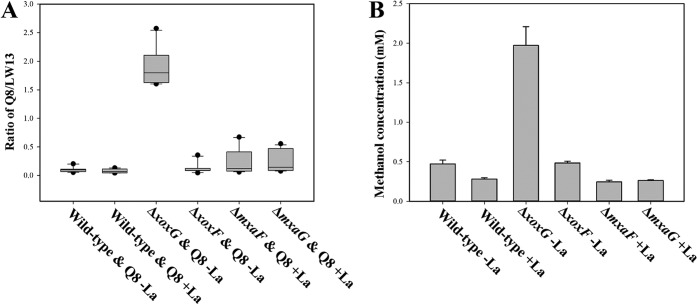
Correlation between ratios of populations of M. methylotrophus Q8 and *Methylomonas* sp. LW13 in synthetic communities fed methane and excreted methanol concentration. (A) Range of ratios of M. methylotrophus Q8 to *Methylomonas* sp. LW13 after the second, third, fourth, and fifth transfer of the coculture. (B) Concentration of methanol excreted by pure cultures of different *Methylomonas* sp. LW13 strains.

As methanotrophs are known to spill methanol and feed satellite communities of nonmethanotrophic methylotrophs and as it has been recently suggested that the genes for alternative MDH enzymes are subjected to regulation in response to communal living (14, 15), we tested whether the community function was affected by mutations in the Xox and Mxa systems. Synthetic communities were constructed of different strains of *Methylomonas* sp. LW13 and Methylophilus methylotrophus Q8, a model nonmethanotrophic methylotroph, and these strains were fed methane as the sole carbon source. Every 48 h, cultures were diluted to an optical density at 600 nm (OD_600_) of less than 0.1 in fresh medium, with a total of five transfers employed in this experiment (Fig. S4), and cells of *Methylomonas* sp. LW13 and M. methylotrophus Q8 were counted by flow cytometry at the end of each growth period (Fig. S5). Wild-type *Methylomonas* sp. LW13 established stable communities with M. methylotrophus Q8 in both the presence and absence of La (Fig. S5). The Δ*xoxF* mutant grown in the absence of La revealed a similar pattern in terms of growth rate or community composition (approximately 0.1 to 1.0 ratio of M. methylotrophus Q8 to *Methylomonas* sp. LW13, judged by cell counts) (Fig. S5). In the presence of La, growth of the Δ*xoxF* mutant was originally repressed, due to the La switch (see above), but it resumed gradually, after approximately 48 h, due to the appearance of suppressor mutations (see below), and the abundance of M. methylotrophus Q8 also gradually increased (Fig. S5). Δ*mxaF* and Δ*mxaG* mutants supported somewhat higher populations of M. methylotrophus Q8 (0.4:1.0 to 0.5:1.0) when cultivated in the presence of La, while the Δ*xoxG* mutant, at an overall low cell density, supported the highest population of M. methylotrophus Q8 (approximately 2.0 to 1.0), when cultivated in the absence of La (Fig. S5). We measured concentrations of methanol in the media after pure cultures of different strains of *Methylomonas* sp. LW13 reached stationary phase (Fig. 4B). The concentration of the excreted methanol was the highest for the Δ*xoxG* mutant, suggesting a leaky phenotype and thus potentially explaining the highest ratio of M. methylotrophus Q8 to *Methylomonas* sp. LW13 in the respective coculture (Fig. 4A).

10.1128/mBio.02430-17.4 FIG S4  Growth curves for synthetic communities consisting of the wild type or mutant strains of *Methylomonas* sp. LW13 and M. methylotrophus Q8. OD_600_ data were recorded over 48 h, followed by dilution and transfer of cultures into fresh medium. Note that the XoxF mutant starts growing in the presence of La after approximately 48 h, due to the appearance of suppressor mutants. Headspace (75% air/25% methane) was refreshed every 12 h. Data represent means from three replicates ± standard deviations. Download FIG S4, TIF file, 1.8 MB. Copyright © 2018 Zheng et al. 2018 Zheng et al. This content is distributed under the terms of the Creative Commons Attribution 4.0 International license.

10.1128/mBio.02430-17.5 FIG S5  Cell abundance dynamics of individual species in synthetic communities. Individual abundances of *Methylomonas* sp. LW13 and M. methylotrophus Q8 were measured every 48 h before dilution/transfer. Data represent means from three replicates ± standard deviations. Download FIG S5, TIF file, 2.7 MB. Copyright © 2018 Zheng et al. 2018 Zheng et al. This content is distributed under the terms of the Creative Commons Attribution 4.0 International license.

### Analysis of ΔXoxG and ΔXoxF suppressor mutants.

It has been previously demonstrated that, in the presence of Ln, ΔXoxF mutants quickly accumulate suppressor mutations, resulting in a broken Ln switch (8). Indeed, we also observed quick transition of ΔXoxF mutants from being unable to grow in the presence of Ln to wild-type growth, manifested by only 48-h delay in growth in liquid cultures (Fig. S4), or by the appearance of single colonies on plates containing Ln. After selection, such suppressors could grow at the wild-type rate in the presence or absence of Ln (Fig. S6A) and express wild-type levels of MxaFI (Fig. S6C). In Methylomicrobium buryatense, the causative mutation resulting in the broken Ln switch has been detected in the *mxaY* gene, encoding a histidine kinase that is involved in inverse regulation of transcription from both *mxaF* and *xoxF* (8). A known response regulator for MxaY, MxaB has also been implicated in regulation of *mxaF* and *xoxF* expression (8). Thus, we tested several XoxF suppressor mutants for mutations in *mxaY* and *mxaB* by PCR amplifying and sequencing the genes in question. We indeed identified mutations in *mxaY* (for example, a mutation resulting in a substitution of the acidic glutamic acid 37 by the basic lysine [data not shown]). However, one of the tested suppressor mutants did not contain any mutations in *mxaY* or *mxaB*. This mutant also revealed a specific phenotype. Instead of a smooth biofilm formed by wild-type *Methylomonas* sp. LW13, it formed a rough biofilm (result not shown). The genome of this mutant was resequenced and analyzed for the presence of potential suppressor mutations. A total of four mutations were identified and subsequently confirmed via PCR analysis (Table 2). These mutations were in genes encoding a flavoprotein, a function essential for cell membrane formation, and in two different exosortase genes that are implicated in extrusion of exopolysaccharides by Gram-negative bacteria (24). The functions of the latter two genes are consistent with the phenotype of the mutant, forming a different type of biofilm compared to wild-type strain. However, it is not immediately clear from these predictions how the change in biofilm properties relates to the broken Ln switch, and testing the effect of each individual mutation will be pursued in the future.

10.1128/mBio.02430-17.6 FIG S6  Growth curves of representatives of suppressor mutants in XoxF (A) and XoxG (B) grown with or without 30 μM La. (C, top) In-gel activity staining reveals expression of MxaFI-type, but not XoxF-type, MDH. (Bottom) Specific MDH activities are similar between original mutant and suppressor variants. Data represent means from three replicates ± standard deviations. Download FIG S6, TIF file, 1.8 MB. Copyright © 2018 Zheng et al. 2018 Zheng et al. This content is distributed under the terms of the Creative Commons Attribution 4.0 International license.

**TABLE 2:** Mutations detected in suppressor mutants via genome resequencing

Scaffold ID^a^	Position	Variant	Type	Locus tag	IMG product name^b^
XoxG suppressor mutants					
U737DRAFT_scaffold00012	20996	TCC → TAC	Missense variant	U737DRAFT_03233	Two-component system, OmpR family, sensor histidine kinase QseC
U737DRAFT_scaffold00009	145703	TGG → GGG	Missense variant	U737DRAFT_02788	AAA^+^-type ATPase, SpoVK/Ycf46/Vps4 family
U737DRAFT_scaffold00001	473441	GAG → TAG	Stop gained	U737DRAFT_00415	Outer membrane receptor proteins, mostly Fe transport
U737DRAFT_scaffold00007	102811	TAT → TAA	Stop gained	U737DRAFT_02300	Flagellum-specific ATP synthase

XoxF suppressor mutants with the rough biofilm phenotype					
U737DRAFT_scaffold00015	31983	GAT → AAT	Missense variant	U737DRAFT_03712	Riboflavin synthase alpha chain
U737DRAFT_scaffold00024	42129	CAT → CAA	Missense variant	U737DRAFT_04473	Acyl-[acyl-carrier-protein]-phospholipid *O*-acyltransferase/long-chain-fatty-acid– [acyl-carrier-protein] ligase
U737DRAFT_scaffold00019	1330	C → CATGCA CCAAAT	Indel	U737DRAFT_04087	Transmembrane exosortase (Exosortase_EpsH)
U737DRAFT_scaffold00030	95	C → CGTTAGCG GATTTGGCTGGTT	Indel (intergenic region)	U737DRAFT_04704	Exosortase A

a Scaffold ID, scaffold identification number.

b IMG, Integrated Microbial Genomes Database (https://img.jgi.doe.gov).

Ln-resistant variants of ΔXoxG mutants were also selected. However, they did not exhibit the wild-type growth rate (Fig. S6B), further supporting the notion of the dual function of XoxG. Remarkably, in ΔXoxG suppressor mutants, only MxaFI MDH was active, and not XoxF (Fig. S6C), suggesting that the broken switch not only reversed expression of *mxaFI*, but that in this case, it also affected either expression or activity of XoxF (Fig. S6C). In contrast to ΔXoxF suppressor mutants, none of the ΔXoxG suppressor mutants contained mutations in either *mxaY* or *mxaB*. This outcome was not surprising, as *xoxG* does not appear to be coregulated with *xoxF* (15). The genomes of five such mutants were resequenced. All five mutants contained identical sets of four mutations (Table 2), suggesting that these mutants were likely clonal. The mutations in ΔXoxG suppressor mutants did not overlap with the mutations in ΔXoxF suppressor mutants, and these mutations were in genes predicted to encode a component of a transcriptional regulatory pair, a protein involved in cell wall synthesis, an outer membrane receptor specific to metal transport, and an ATP synthase. The functions of each of these genes will be interrogated in future studies.

### Heterologous expression of alternative cytochromes in the XoxG mutant.

**FIG 5:**
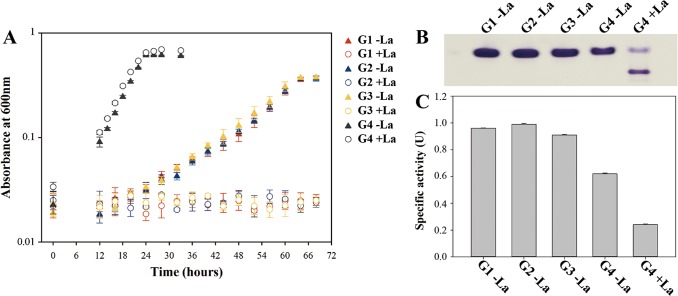
Growth and MDH activities in strains carrying alternative *xoxG* genes. (A) Growth curves of the strains carrying heterologous *xoxG1* (G1), *xoxG2* (G2), *xoxG3* (G3), or *xoxG4* (G4) grown with 30 μM La or without La. (B) In-gel activity staining with extracts of the respective strains. (C) Respective specific MDH activity values.

XoxG of *Methylomonas* sp. LW13 represents a variant of a cytochrome known as XoxG4, specific to gammaproteobacterial methanotrophs and phylogenetically different from other putative XoxG cytochromes (XoxG1, XoxG2, and XoxG3) identified in betaproteobacterial and alphaproteobacterial methylotrophs, respectively (15). This presents a very complicated case, suggesting that, while properties of the MxaFI/MxaG enzyme pairs must be similar in phylogenetically divergent methylotrophs, properties of XoxF/XoxG pairs may be different. We tested whether alternative XoxG proteins (XoxG1, XoxG2, or XoxG3) could functionally substitute for XoxG4 by expressing these alternative variants in the ΔXoxG mutant under the native promoter of *xoxG4*. *xoxG1*, *xoxG2*, and *xoxG3* originated from Methylotenera versatilis strain 7, M. methylotrophus sp. Q8, and *Methylosinus* sp. strain PW1, respectively (see Materials and Methods). For a positive control, we expressed an *xoxG4* homolog from Methylosarcina lacus LW14. Of the resulting complementation constructs, only the *xoxG4* homolog was able to restore wild-type growth of the mutant, while *xoxG1*, *xoxG2*, and *xoxG3* did not change the phenotype of the mutant (Fig. 5A; Table S2). The MDH activity in the *xoxG4*-complemented strain was at wild-type levels with or without La (Fig. 5B), while strains expressing *xoxG1* to *xoxG3* could grow only in the absence of La, at the same rate as that of the original mutant, and these strains exhibited only MxaFI MDH activity (Fig. 5A and B; Table S2).

10.1128/mBio.02430-17.9 TABLE S2  Doubling times for XoxG4 mutant heterologously expressing *xoxG1*, *xoxG2*, *xoxG3*, and *xoxG4*. Download TABLE S2, DOCX file, 0.1 MB. Copyright © 2018 Zheng et al. 2018 Zheng et al. This content is distributed under the terms of the Creative Commons Attribution 4.0 International license.

### Biochemical demonstration of reduction of XoxG by XoxF.

**FIG 6:**
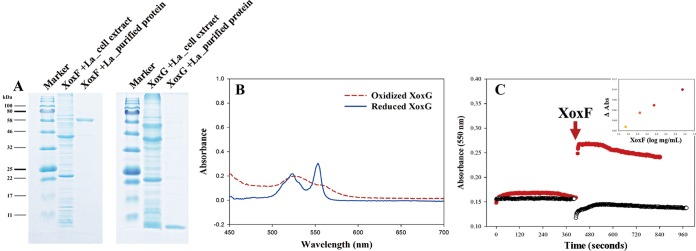
Demonstration of reduction of purified XoxG by purified XoxF. (A) SDS-PAGE analysis of purified XoxF and XoxG, compared to respective cell extracts and protein standards (Marker lanes). (B) UV-visible spectrum of purified XoxG before and after reduction by 5 mM Na_2_S_2_O_4_. (C) Change in absorbance at 550 nm after the addition of purified XoxF to purified XoxG (red lines) or buffer (blue lines). (Inset) XoxF concentration-dependent increase in absorbance at 550 nm.

We tested whether XoxG is indeed a cytochrome *c* and whether it can be directly reduced by XoxF. We expressed and purified the His-tagged versions of these proteins (Fig. 6A and Fig. S7). The purified XoxG, after reduction by dithionite, showed two strong absorption peaks, around 525 and 552 nm (Fig. 6B), as is typical of cytochromes *c* (25, 26), supporting our original assumption. We then recorded absorbance by XoxG, at 550 nm, before and after the addition of the purified XoxF and observed an increase in absorbance, as in the experiments reported with the purified MxaFI and MxaG proteins (25, 26), indicative of the XoxF-dependent reduction of XoxG (Fig. 6C). The increase in the concentration of XoxF correlated with the increase in the absorbance at 550 nm. This experiment demonstrated direct redox interaction between XoxF and XoxG isolated from *Methylomonas* sp. LW13.

10.1128/mBio.02430-17.7 FIG S7  Activity of purified XoxF. (A) In-gel staining and (B) specific MDH activity, using the artificial dye assay. Cells were grown with 30 μM La. Download FIG S7, TIF file, 1.7 MB. Copyright © 2018 Zheng et al. 2018 Zheng et al. This content is distributed under the terms of the Creative Commons Attribution 4.0 International license.

## DISCUSSION

Until the recent discovery of Ln-dependent alcohol dehydrogenases, Ln were assumed to be biologically inert (18), and the roles of these enzymes, as parts of central metabolic pathways, and the physiological role of the “Ln switch” remain enigmatic. It also remains unclear how, in nature, such enzymes could reach high activities and how relevant the repression of the MxaFI-type MDH is, when present under natural conditions. While low micromolar concentrations of Ln have been reported for extreme environments such as hot, acidic mudpots (11), in neutral environments, concentrations of Ln are extremely low (27). Thus, until Ln-concentrating mechanisms are discovered and understood, neither the full potential activity for XoxF nor the complete repression of MxaFI MDH enzymes should be assumed, as we demonstrated in this study by visualizing activities of both enzymes simultaneously, at least in the model we employed and at least with the artificial dye assay. We clearly demonstrate that, even in the presence of Ln, the MxaFI MDH can be active. We also demonstrate that either the MxaFI or XoxF enzyme could be removed and that mutants in either enzyme not only retain potential for wild-type growth, as long as appropriate metals are supplied, but they also maintain their potential for communal function, supporting satellite communities of *Methylophilaceae*, as parts of model synthetic communities. We also confirm the previously noted propensity for fast evolution in the presence of Ln, suggesting the fragility of the Ln switch and highlighting the lack of such selective pressure in nature. Thus, it is likely that a balance between the two types of enzymes, rather than the on/off switch, provide metabolic robustness to natural populations of methanotrophs.

We further demonstrate a physiological effect of mutation in *xoxG4*, a recently identified gene. The complex phenotype caused by the mutation in this gene, its independent chromosomal positioning, along with the lack of transcriptional regulation by Ln strongly suggest a secondary function for XoxG4, which may also be in methanol oxidation. We also obtained preliminary data for alternative cytochromes, XoxG1, XoxG2, and XoxG3, encoded by nongammaproteobacterial methylotrophs, not being able to substitute for XoxG4, potentially pointing to differences in redox biochemistry of different XoxF/XoxG pairs. Finally, we obtained biochemical confirmation for XoxG4 being a true cytochrome *c*, based on its spectral analysis, and demonstrated that it could be directly reduced by XoxF.

Overall, while the data presented here provide new insights and expand our understanding of Ln-dependent methanol oxidation, they also further point to the complex nature of the biochemistry catalyzed by different Xox systems and warrant further investigation, employing alternative models representing alternative phylogenetic backgrounds.

## MATERIALS AND METHODS

### Strains and growth conditions.

*Methylomonas* sp. strain LW13 (28) was cultivated in the nitrate mineral salts (NMS) medium (23) with 25% methane and 75% air (vol/vol) in the headspace, with shaking at 200 rpm, at 30°C. Methylophilus methylotrophus Q8 (29) was cultivated on solid NMS medium, with 0.5% (vol/vol) methanol as a substrate. Lanthanum(III) chloride (La^3+^; 99.9% trace metals basis, Sigma-Aldrich) was supplied at concentrations of 0.03 to 60 μM to some of the cultures, as per experimental design. All the glassware employed was acid washed for 24 h in 1 M hydrochloric acid before use, to remove trace amounts of lanthanum adhering to the glass.

### Genetic manipulations.

All genetic manipulations were achieved through electroporation of the assembled PCR amplification-based constructs into the cells of *Methylomonas* sp. LW13, followed by selection of chromosomal recombinants, as previously described (30). Briefly, for the gene knockouts, the kanamycin resistance gene cassette (amplified from the plasmid pCM433 [31]) and the two regions flanking the gene of interest were assembled by fusion PCR (32). The assembled fragment was further amplified using nested primers at a high annealing temperature (32). The resulting product was purified by the QIAquick PCR purification kit (Qiagen), and the product was directly electroporated into *Methylomonas* sp. LW13. After electroporation, cells were precultivated overnight in liquid medium without kanamycin, after which cells were harvested and transferred onto plates containing 50 μg/ml kanamycin. Single colonies were selected, and the desired mutation was verified by sequencing a respective PCR-amplified fragment of the chromosomal DNA. For the heterologous expression of *xoxG* genes, the assembled constructs contained the gene of interest, the kanamycin resistance gene cassette, and the two regions flanking *xoxG*. The heterologously expressed genes belonged to Methylotenera versatilis 7 (*xoxG1*; K370DRAFT_1204 [22]), M. methylotrophus Q8 (*xoxG2*; GQ52DRAFT_0576 [22]), *Methylosinus* sp. strain PW1 (*xoxG3*; K369DRAFT_3505 [33]), and Methylosarcina lacus LW14 (*xoxG4*; MetlaDRAFT_0341 [22]). For protein expression and purification, His tags encoding six histidine residues were added to the 3′ terminus of the target gene.

### Purification of XoxG and XoxF.

Cultures were grown in the presence of 30 μM La to a final OD_600_ of 0.7 to 0.8. Cells from 1 liter of culture were harvested by centrifugation at 5,000 rpm for 15 min at 4°C, and cell pellets were quickly frozen in liquid nitrogen and stored at −80°C. Cell pellets were resuspended in 100 mM Tris-HCl (pH 9.0) buffer and passed twice through a French pressure cell (Sim-Aminco) at 10^8^ Pa. Cell lysates were centrifuged at 13,000 rpm for 30 min at 4°C to remove cell debris. Cell extracts were mixed with 5 volumes of the following buffer: 100 mM Tris-HCl (pH 9.0), 150 mM NaCl, 1 mM methanol, 5 mM imidazole, and shaken for 10 min at 100 rpm at 4°C. Pierce protease inhibitor (Thermo Fisher Scientific) was added to the mixture before shaking. Proteins were purified by metal ion affinity chromatography as follows. The above mixtures were loaded onto the equilibrated 2-ml Ni-nitrilotriacetic acid superflow resin (Qiagen). The resins with the attached His-tagged proteins were washed sequentially with 3 ml of the buffer containing 5 mM imidazole and the buffer containing 50 mM imidazole. Protein elution was achieved using 2 ml of the buffer containing 250 mM imidazole. The samples were then desalted, and the buffer was replaced by dilution with 100 mM Tris-HCl (pH 9.0) buffer (20-fold, repeated five times), followed by concentration by centrifugation in 10-kDa-cutoff dialysis tubes (Millipore, Billerica, MA), in the case of XoxG and in 50-kDa cutoff tubes in the case of XoxF until the calculated concentration of imidazole reached less than 1 μM. The purified proteins were analyzed by separation in a 12% SDS-denaturing polyacrylamide gel, followed by Coomassie blue staining.

### Methanol dehydrogenase assay.

A standard assay employing artificial dyes (34) was used to measure MDH activity (100 mM Tris-HCl [pH 9.0], 45 mM NH_4_Cl, 5 mM methanol, 1 mM phenazine methosulfate [PMS], 0.1 mM 2,6-dichlorophenolindophenol [DCPIP]). Assays were performed at room temperature (25°C) in plastic cuvettes, and the reduction of DCPIP was monitored spectrophotometrically at 600 nm. Protein concentration was measured by the bicinchoninic acid assay (Sigma-Aldrich, St. Louis, MO).

For staining in the gel, cell extracts or purified protein samples (approximately 3 mg/ml protein) were mixed with native sample buffer (Bio-Rad) and subjected to native gradient gel electrophoresis (4 to 25% Mini-PROTEAN TGX precast protein gels; Bio-Rad). Activity staining was done essentially as previously reported (35). Briefly, a gel was immersed in a solution of 100 mM Tris-HCl (pH 9.0), 45 mM NH_4_Cl, 1 mM nitroblue tetrazolium, 1 mM PMS, and 5 mM methanol and incubated at 37°C in the dark for 10 to 30 min.

### Synthetic community manipulations.

*Methylomonas* sp. LW13 and M. methylotrophus Q8 were pregrown on methane in liquid and on methanol on plates (to avoid carryover of methanol), respectively, and then mixed together in glass tubes (28-ml volume) in 6 ml of the NMS medium. The headspace composition was adjusted to 75% air/25% methane and refreshed every 12 h according to the following scheme: (i) flushing with air for 40 s; (ii) equalizing pressure by removing the excess air with a syringe; (ii) removing 5.5 ml of headspace and then adding 5.5 ml of methane. After 48 h, each culture was diluted to an OD_600 _of less than 0.1 in fresh medium, and cultures were incubated as described above. A total of five transfers were carried out. At the time of each transfer, samples were taken for flow cytometric cell counting, essentially as described before (14). Briefly, 900-μl samples were fixed immediately with 100 μl of a fixation solution (1.6% glutaraldehyde, 0.1% paraformaldehyde [vol/vol]). For each analysis, 3 to 5 μl of fixed sample was mixed with 10 μl of SYBR green dye (Thermo Fisher Scientific; 1:100 in 100% dimethyl sulfoxide) and 0.22-μm-filtered NMS medium to a final volume of 830 μl. Samples were incubated for 30 min in the dark, at room temperature. Cells were counted using a CyFlow space flow cytometer (Partec), triggering on green fluorescence. The detailed parameters were as follows: measured parameters side scatter (SSC), forward scatter (FSC), and green fluorescence were displayed in log 3 or log 4; flow rate, 4 μl/s; particle analysis rate, below 1,000 particles/s. Cells of *Methylomonas* sp. LW13 and M. methylotrophus Q8 were counted separately on the basis of the significant difference in their size (14) (Fig. S1).

### Methanol detection.

Methanol secreted by *Methylomonas* sp. LW13 was measured using a commercial methanol assay kit (colorimetric; BioVision).

### Genome resequencing.

Δ*xoxG* and Δ*xoxF* suppressor mutants were isolated from plates supplemented with La. Genomic DNA was isolated using the GeneJET genomic DNA purification kit (Thermo Fisher Scientific, USA). DNA was resequenced using the Illumina technology, obtaining paired-end 150-bp sequences for the 300-bp inserts by GENEWIZ (USA). Raw data were trimmed with Trimmomatic (v2.4.0), and the PCR duplicates were removed by FastUniq (v1.1). High-quality data were mapped to the reference genome using bowtie2 (v2.3.0). The mapped data were called by GATK (v3.7) and Samtools (v1.4) for variant analysis, including single nucleotide polymorphisms (SNP) and indels. The mapped reads were also processed with breseq (0.31.1). Finally, variant results were summarized and filtered by GATK, Samtools, and breseq.

### RNA-seq analysis.

RNA was extracted from the wild-type strain culture and from Δ*xoxG* mutant culture of *Methylomonas* sp. LW13 grown without lanthanum. Cells were collected in the logarithmic phase of growth (OD_600_ of ~0.5 for the wild type and ~0.3 for the Δ*xoxG* mutant). Cells were lysed by bead beating in QIAzol (Qiagen) and mixed with chloroform. After centrifugation, the colorless upper aqueous phase was precipitated by adding 100% ethanol to a final concentration of 70%. RNA was purified by using the miRNAeasy minikit (Qiagen). The contaminating DNA was removed with Turbo DNase (Ambion), and RNA was recovered using RNeasy MinElute cleanup kit (Qiagen). The purified RNA was checked for DNA contamination using 16S rRNA gene fragment PCR amplification assay. Samples were stored at −80°C before sequencing. Paired-end 150-bp reads were obtained using the HiSeq 2500 technology by GENEWIZ (USA). Raw reads were trimmed by Trimmomatic (version 0.36), and these were aligned with the reference genome using Bowtie2 (version 2.3.4). The alignments were postprocessed into sorted BAM files with Samtools (version 0.1.19). Reads were attributed to open reading frames (ORFs) using the htseq count tool from the HTseq. Differential abundance analysis was performed with DESeq2 (version 1.2.10).
