# Supplementary figures and images for: Physiological Effect of XoxG(4) on Lanthanide-Dependent Methanotrophy
Source: mBio. 2018 Mar 27;9(2):e02430-17. doi: 10.1128/mBio.02430-17 (PMC5874918; doi:10.1128/mBio.02430-17)

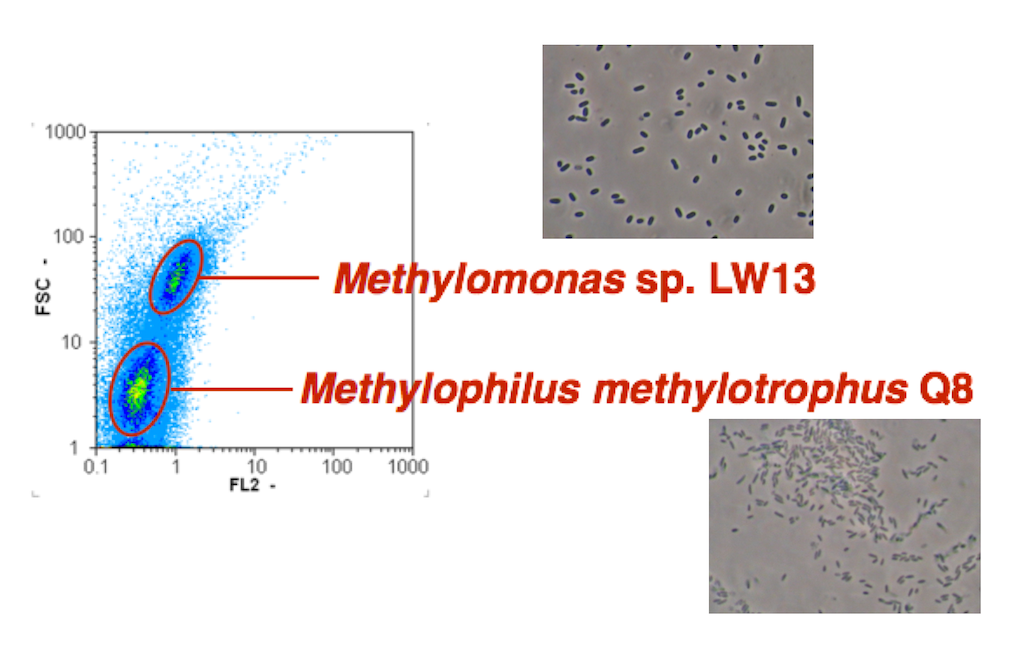

Supplement: FIG S1 [file mbo002183802sf1.tif]

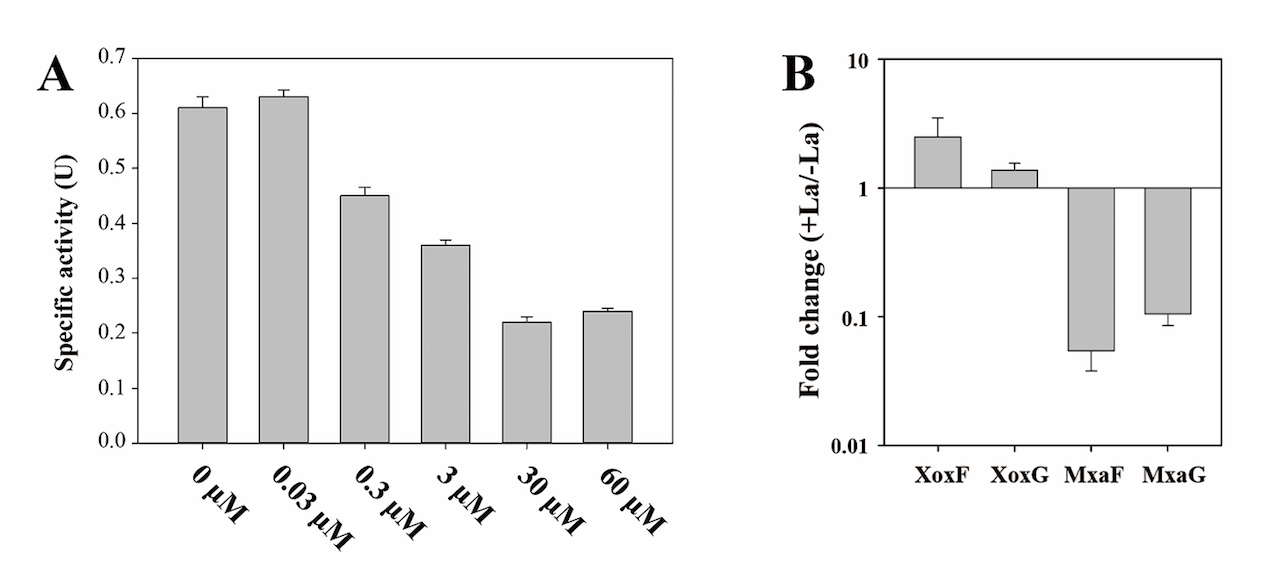

Supplement: FIG S2 [file mbo002183802sf2.tif]

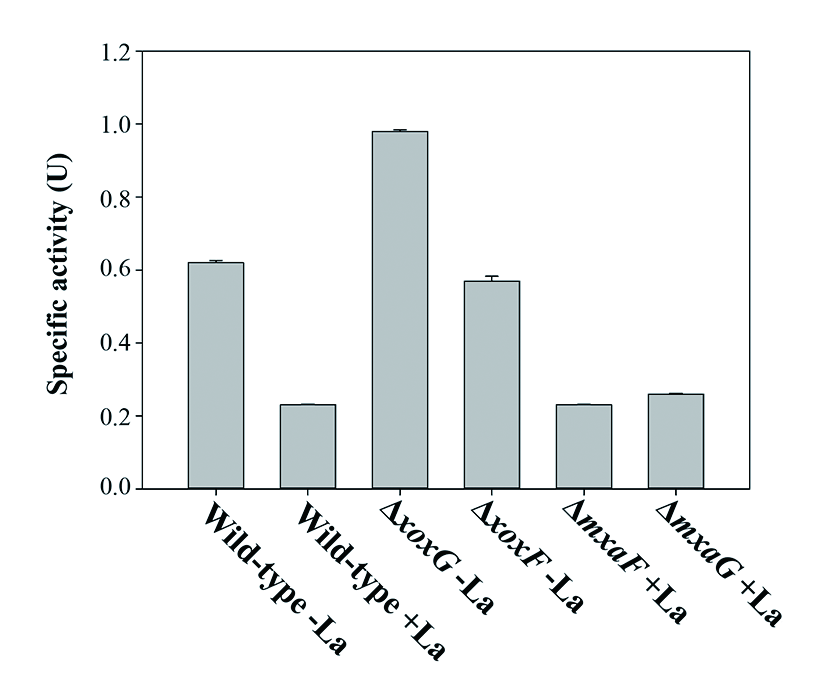

Supplement: FIG S3 [file mbo002183802sf3.tif]

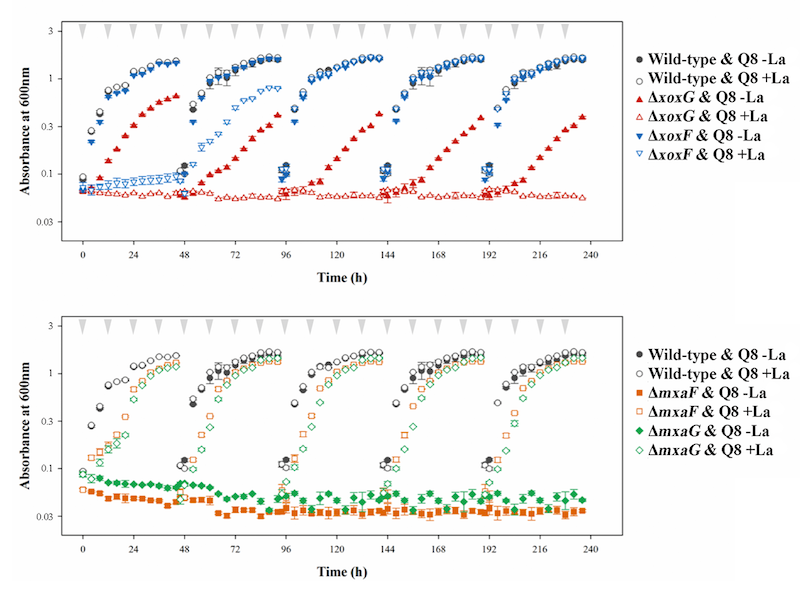

Supplement: FIG S4 [file mbo002183802sf4.tif]

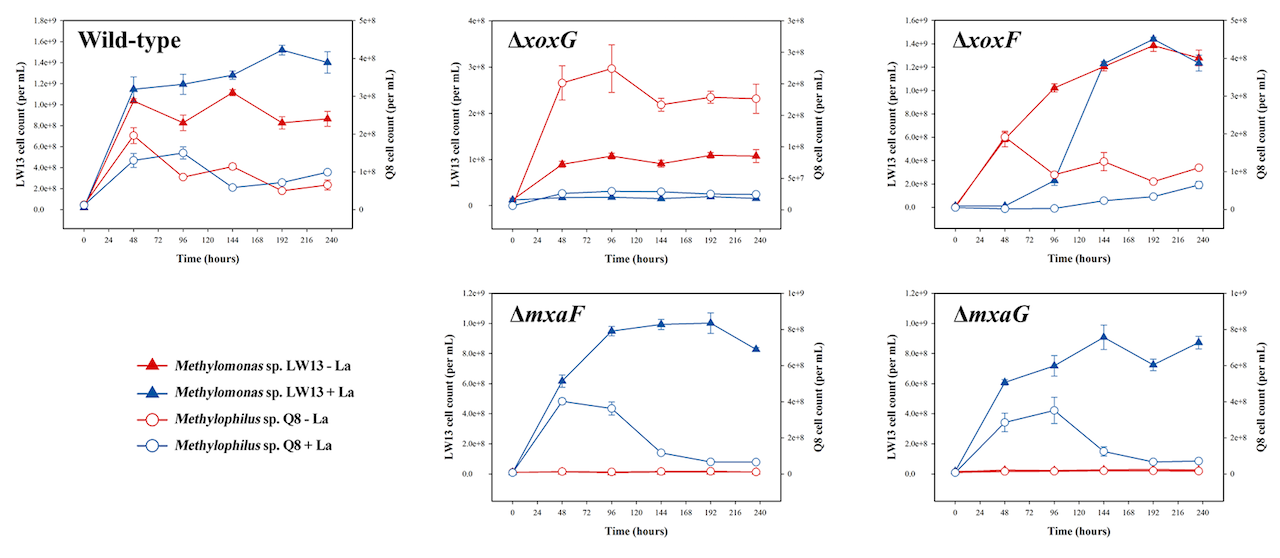

Supplement: FIG S5 [file mbo002183802sf5.tif]

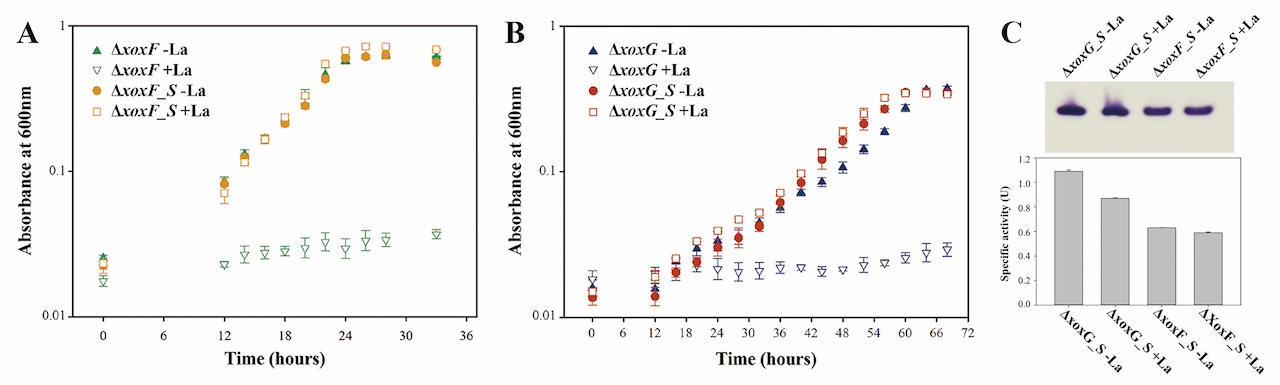

Supplement: FIG S6 [file mbo002183802sf6.tif]

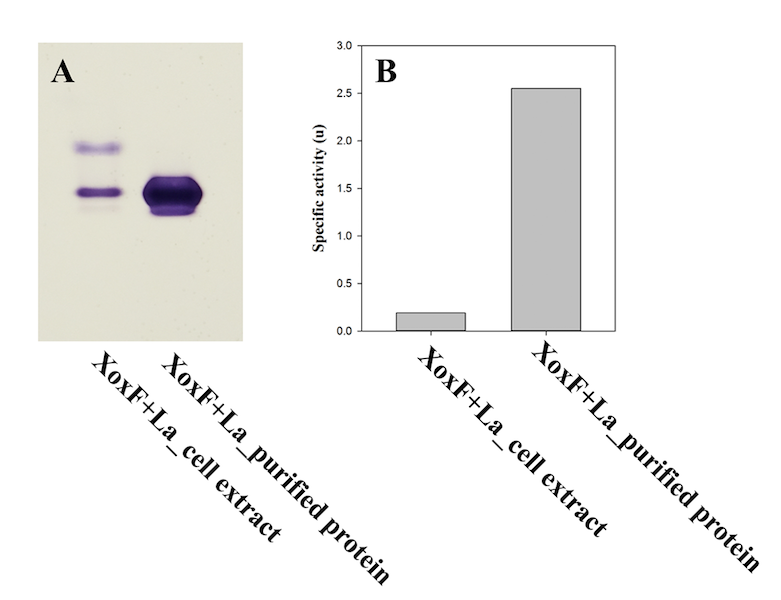

Supplement: FIG S7 [file mbo002183802sf7.tif]
