# Supplementary material for: Physiological Effect of XoxG(4) on Lanthanide-Dependent Methanotrophy
Source: mBio. 2018 Mar 27;9(2):e02430-17. doi: 10.1128/mBio.02430-17 (PMC5874918; doi:10.1128/mBio.02430-17)
Supplement: TABLE S1 [file mbo002183802st1.docx]

**Table S1.** Differential gene expression between wild-type and Δ*xoxG* strains

| Gene_locus_ID | Product_name | Wild type-1 | Wild type-2 | Δ*xoxG* -1 | Δ*xoxG* -2 |
| --- | --- | --- | --- | --- | --- |
| U737DRAFT_00206 | Ribosomal protein L16 Arg81 hydroxylase, contains JmjC domain | 169.62 | 177.85 | 17.85 | 16.10 |
| U737DRAFT_00207 | Hypothetical protein | 104.42 | 99.96 | 9.00 | 5.89 |
| U737DRAFT_00208 | Hypothetical protein | 97.95 | 84.13 | 6.30 | 3.86 |
| U737DRAFT_00209 | Hypothetical protein | 95.82 | 110.76 | 8.41 | 3.69 |
| U737DRAFT_00210 | Hypothetical protein | 95.53 | 85.39 | 9.54 | 7.70 |
| U737DRAFT_00357 | Cysteine desulfurase | 88.34 | 107.53 | 1028.26 | 1028.58 |
| U737DRAFT_00500 | outer membrane Transport energization protein ExbB | 83.15 | 92.46 | 8.12 | 6.65 |
| U737DRAFT_00521 | Bacteriocin-type signal sequence-containing protein | 37.31 | 34.72 | 314.44 | 275.89 |
| U737DRAFT_00932 | Chaperonin GroES | 118.50 | 111.83 | 1431.15 | 1100.91 |
| U737DRAFT_00933 | Chaperonin GroEL | 497.04 | 404.76 | 5341.35 | 5083.22 |
| U737DRAFT_01086 | Hypothetical protein | 239.93 | 262.33 | 26.84 | 30.92 |
| U737DRAFT_01497 | hemerythrin | 2870.14 | 2132.66 | 48.74 | 50.19 |
| U737DRAFT_01514 | Hypothetical protein | 394.97 | 479.66 | 5.14 | 2.22 |
| U737DRAFT_01588 | Sel1 repeat-containing protein | 5.47 | 4.05 | 41.53 | 44.24 |
| U737DRAFT_01589 | General secretion pathway protein D | 10.23 | 8.66 | 91.45 | 95.09 |
| U737DRAFT_01607 | Phosphoenolpyruvate carboxylase, type 1 | 21.35 | 20.03 | 252.22 | 262.13 |
| U737DRAFT_01651 | RNA polymerase primary sigma factor | 106.25 | 115.85 | 1157.09 | 1223.31 |
| U737DRAFT_01756 | Vitamin B12 transporter | 66.21 | 68.62 | 959.99 | 988.65 |
| U737DRAFT_02361 | Transcriptional regulator, BadM/Rrf2 family | 59.95 | 65.19 | 528.69 | 499.01 |
| U737DRAFT_02362 | Fe-S cluster assembly protein SufB | 102.37 | 127.22 | 1294.17 | 1290.89 |
| U737DRAFT_02363 | Fe-S cluster assembly ATP-binding protein | 101.79 | 113.33 | 1050.77 | 1099.13 |
| U737DRAFT_02364 | Fe-S cluster assembly protein SufD | 126.87 | 147.02 | 1238.65 | 1274.53 |
| U737DRAFT_02366 | Cysteine desulfurase / selenocysteine lyase | 51.45 | 63.11 | 691.66 | 705.79 |
| U737DRAFT_02510 | Hypothetical protein | 9.58 | 10.02 | 105.90 | 78.46 |
| U737DRAFT_02821 | Hypothetical protein | 27.12 | 25.38 | 244.51 | 222.74 |
| U737DRAFT_03213 | Methyl-accepting chemotaxis protein | 1305.94 | 1315.48 | 109.96 | 130.13 |
| U737DRAFT_03214 | Purine-binding chemotaxis protein CheW | 803.47 | 792.05 | 80.22 | 84.00 |
| U737DRAFT_03878 | Membrane fusion protein | 57.21 | 64.54 | 673.65 | 730.32 |
| U737DRAFT_03879 | Hypothetical protein | 548.19 | 504.41 | 7738.64 | 8265.89 |
| U737DRAFT_03880 | Hypothetical protein | 219.13 | 203.48 | 3259.40 | 3047.52 |
| U737DRAFT_03881 | Hypothetical protein | 47.44 | 50.56 | 645.65 | 496.83 |
| U737DRAFT_04004 | Hypothetical protein | 482.26 | 464.13 | 4096.19 | 3878.46 |
| U737DRAFT_04005 | Hypothetical protein | 481.37 | 441.19 | 4313.58 | 4370.12 |
| U737DRAFT_04231 | Hypothetical protein | 1542.44 | 1698.69 | 89.31 | 74.23 |
| U737DRAFT_04392 | Anti-sigma-28 factor, FlgM family | 371.57 | 464.83 | 47.58 | 47.00 |
| U737DRAFT_04803 | Methyl-accepting chemotaxis protein | 850.98 | 953.83 | 104.80 | 107.63 |

Differential gene expression was normalized by TPM (Transcripts Per Kilobase of exon model per Million mapped reads), and selected by *P*<0.05 and fold change >8.
