# Supplementary material for: Physiological Effect of XoxG(4) on Lanthanide-Dependent Methanotrophy
Source: mBio. 2018 Mar 27;9(2):e02430-17. doi: 10.1128/mBio.02430-17 (PMC5874918; doi:10.1128/mBio.02430-17)
Supplement: TABLE S2 [file mbo002183802st2.docx]

**Table S2.** Doubling times of XoxG4 mutant heterologously expressing *xoxG1, xoxG2, xoxG3* and *xoxG4*

| Strain | Doubling time (h) | | |
| --- | --- | --- | --- |
|  | Without La | With La |  |
| Δ*xoxG* | 11.86 ± 1.01 | -- |  |
| G1 | 12.94 ± 0.92 | -- |  |
| G2 | 14.18 ± 2.44 | -- |  |
| G3 | 12.15 ± 1.69 | -- |  |
| G4 | 4.15 ± 0.30* | 4.07 ± 0.17* |  |

Doubling times represent the means for three replicates with standard deviations, and they were calculated from four time points during the exponential phase of growth. La was supplied at 30 μM. Asterisk indicates statistically highly significant change in growth rate compared to other strains (*P*<0.001).
